# Key programmatic and policy considerations for introducing multipurpose prevention (MPT) methods: reflections from healthcare providers and key stakeholders in South Africa

**DOI:** 10.3389/frph.2024.1249750

**Published:** 2024-02-20

**Authors:** Alison Kutywayo, Paballo Mataboge, Nqaba Mthimkhulu, Catherine E. Martin, Lorrein S. Muhwava, Mbali Mazibuko, Nthabiseng Makalela, Khanyiswa Kwatsha, Vusile Butler, Saiqa Mullick

**Affiliations:** Wits RHI, University of the Witwatersrand, Johannesburg, South Africa

**Keywords:** healthcare providers, multipurpose prevention methods, MPTs, South Africa, multipurpose prevention technologies, policy considerations, programmatic considerations

## Abstract

**Introduction:**

Multipurpose prevention technologies (MPTs) simultaneously prevent HIV, other sexually transmitted infections, and/or unintended pregnancy. Key gatekeepers, [healthcare providers (HCPs) and key stakeholders] require proactive engagement before product implementation. This manuscript identifies HCP demand creation strategies, key stakeholder considerations for the adoption of MPTs in South Africa.

**Methods:**

Formative research was conducted in three districts in three South African provinces (July to November 2022). Nurses initiating oral PrEP at facility and mobile study sites participated in 4-hour participatory workshops, exploring HIV prevention, including MPTs, demand creation strategies, and preferred MPTs training packages. Activities were observed, transcribed, and thematically analysed. Five online in-depth interviews (IDIs) with Key informants (KIs) (National/district programme implementers and technical leads) and one in person, exploring key programmatic and policy considerations for MPT adoption. IDIs were approximately 40 min long, audio recorded, transcribed, and thematically analysed.

**Results:**

Twenty-one Professional Nurses completed workshops: 19 female. Six IDIs were conducted with 4 Facility Managers, 1 NDoH representative and 1 DoH Provincial Deputy Director. All participants were females, aged 30–60+ years with >10 years' in SRH/HIV policy/advocacy/research. Community conversations and information at the clinic were the best MPT demand creation methods among HCPs. KIs identified five considerations for future MPT implementation: HCP training; demand creation and messaging; existing PrEP policy amendments; preparing users for additional choice; and sustaining MPT provision.

**Conclusion:**

Contraceptive implant and oral PrEP implementation lessons learned should be proactively considered when preparing for MPT introduction. HCP training and demand creation are of particular importance before MPT introduction.

## 1 Introduction

In South Africa, HIV prevalence among young women in their early 20's is 9.1%, three times more than that of their male counterparts (1, 2). Adolescent girls and young women (AGYW) (15–24 years) have the highest HIV incidence (1.51%) (3). In addition to HIV, there is also a substantial burden of unintended pregnancy and sexually transmitted infections (STIs) in this population group. A Unitaid-funded PrEP Project working across four South African sites since 2018, led by Wits RHI, found that a third of AGYW were not using contraception at their first visit, when they were initiated on oral PrEP (4). In the same study, among a sub-set of PrEP clients tested for STIs, approximately a third had a curable STI (5).

Since 2017, oral Pre-Exposure Prophylaxis (PrEP) has been made available in South Africa seeking to prevent HIV infection (6). Integrated into sexual and reproductive health (SRH) services in 3034 primary healthcare public health facilities nationwide, 658 885 individuals had been initiated on PrEP, as of July 2022 (7). Effective use of oral PrEP has been proven to reduce HIV infections, however, maintaining adequate continued use remains a significant challenge for HIV prevention (8, 9). Barriers to effective PrEP use include fear of disclosing PrEP use to partners, daily pill fatigue, and stigma associated with pill taking when PrEP is misidentified as antiretroviral therapy (ART) (9–11).

PrEP programmes have shown an unmet need among AGYW, not only for HIV prevention but also for contraception (4, 12) and sexually transmitted infection (STI) diagnosis and management (13). In 2014, the subdermal contraceptive implant was introduced in South Africa, expanding the contraceptive method mix and availability of long-acting reversible methods in the public sector (14). However, data shows almost a 50% drop in insertions year on year (15), and the media reported that early implant removals were common and healthcare-provider resistance to women wanting to remove it was a challenge (16).

Multipurpose prevention technologies (MPTs) are products designed to simultaneously prevent HIV, other STIs, and/or unintended pregnancy (17–19). MPTs may address some of the barriers that currently exist around PrEP (20) and contraception use (2, 21). There are several MPTs in development, ranging from pre-clinical to clinical phase, each adopting a different delivery method (long acting injectables, oral pills, vaginal rings and films, implants, and transdermal compounds) (18). An implant has the potential to offer longer-term, reversible protection; however, noting that the contraceptive implant is not widely used in South Africa, and the challenges with its initial roll out in the country, early engagement with stakeholders is critical. The hypothetical MPTs of interest in this study were the one-year or two-year non-biodegradable, biodegradable and refillable subcutaneous implants, providing simultaneous prevention against HIV and pregnancy. These MPTs are not currently available.

Healthcare providers (HCPs) are key gatekeepers at various levels of health system governance and healthcare service provision (22). In South Africa, HCP attitudes are a known barrier to healthcare uptake (23) but evidence shows that actively engaging them when planning and implementing major health programmes improves both health outcomes and the health system (24, 25). Lanham et al. (2011) researched 113 HCPs at 36 public, private, and non-governmental health facilities in South Africa, Zimbabwe and Kenya that were offering PrEP. HCPs reported that it was challenging to deliver PrEP and SRH services to girls <18 years compared to those >18 because they had negative attitudes about adolescent girls being sexually active. In their review of uptake and early removals of the contraceptive implant in South Africa, Adeagbo et al. (26) found that contraceptive method preferences influenced HCP prescribing patterns for adolescents. Formative research with key stakeholders is key prior to product development and introduction to gain an informed understanding of local populations, socio-cultural norms and practices, and local perceptions, to ensure the introduction of the new products meets the needs and priorities of the end-users (22, 25).

This manuscript presents some of the results from formative research to determine HCP training needs and demand creation strategies to deliver and support the adoption of new MPT methods. We also sought to understand the policy and programmatic considerations for adopting alternative PrEP options from the perspective of key South African stakeholders (facility and district managers and technical leads). This research was part of a larger formative research project aiming to estimate the potential uptake of a hypothetical MPT implant to inform product development (23, 27).

## 2 Methods

### 2.1 Study design and setting

This component was a qualitative, descriptive study conducted between July and November 2022, through four workshops with 21 HCPs, and six in-depth interviews (IDIs) with six Key informants (KIs). Workshops were conducted using participatory action research (PAR). PAR emphasizes social change and transformation, active collaboration through participation between the researcher and members of the system, and iterative cycles of action and reflection to address practical concerns (28, 29). PAR is influenced by understanding that the culture, history, and local context of end-users is embedded in social relationships (30).

The study was carried out in three districts in three South African provinces (Tshwane District, Gauteng Province; OR Tambo District, Eastern Cape Province; and King Cetshwayo District, KwaZulu-Natal Province) leveraging the existing geographical footprint of Wits RHI implementation science projects, which are introducing oral PrEP. Each of the sites is in areas with a high HIV burden, with antenatal HIV prevalence ranging from 23% in Tshwane to 35% in OR Tambo (31). KIs were recruited from these three districts as well as from the National Department of Health.

### 2.2 Study population, recruitment, and sampling

The population for this component of the study comprised of HCPs and KIs.

#### 2.2.1 Healthcare providers

HCPs included in the study workshops were nurses, providing oral PrEP, contraceptive, and SRH services at facility-based and community-based mobile study sites. HCPs were purposively recruited by trained fieldworkers. Those interested were provided with a study overview and what their participation entailed. Willing HCPs were then given the study information sheet and consent form, to review and bring to the workshop. HCPs provided fieldworkers with their contact details, to enable the study team to communicate the day, time, and venue, of the workshop.

#### 2.2.2 Key informant IDIs

In-depth interviews were conducted with KIs, such as Facility Managers of selected study sites, district HIV and AIDS/STI/TB unit (HAST) managers, and PrEP and family planning technical leads at the National Department of Health (NDoH).

Purposive sampling was used to identify KIs, based on their roles in developing and implementing HIV prevention and SRH programmes. Eleven KIs were initially identified, three from each of the sites and two from a national level, seeking to include individuals working across various geographies and in various roles in the health system. Participants were approached via email and provided written, informed consent for participation and an audio recording of the interview.

### 2.3 Study procedures

#### 2.3.1 Healthcare provider workshops

Participants were provided with information on new HIV prevention products and MPT implants. We sought to explore insights on suitable demand creation strategies and tactics to support the uptake of HIV prevention methods (including new MPTs) and to gather insights on the preferred packages for training clinicians on MPTs. One workshop of approximately 4 h was conducted per province with HCPs, with an average of 7 participants in each group. Workshops were held at a community-based site, convenient for participants. They were facilitated in English by the trained study team.

Each workshop was observed by at least two study team members, using an observation guide, which took the observers through a key set of questions for each activity of the workshop. The guide was used to capture the discussion points, participant questions, and reflections and to document the PAR activities. For example, when HCPs were developing their ideal training package, the observers documented participant responses to the facilitator about why they liked that specific training methodology. As partial participants (32), the observers took part in the interactions of the overall workshop but not in the specific PAR activities.

An overview of existing, and future HIV prevention methods as well as the various MPTs in clinical development was presented. Following this, HCPs participated in two PAR activities, demand creation elections and *izikhokho zegame* (skills & knowledge), which are outlined as follows:

##### 2.3.1.1 Demand creation elections

**Figure 1:**
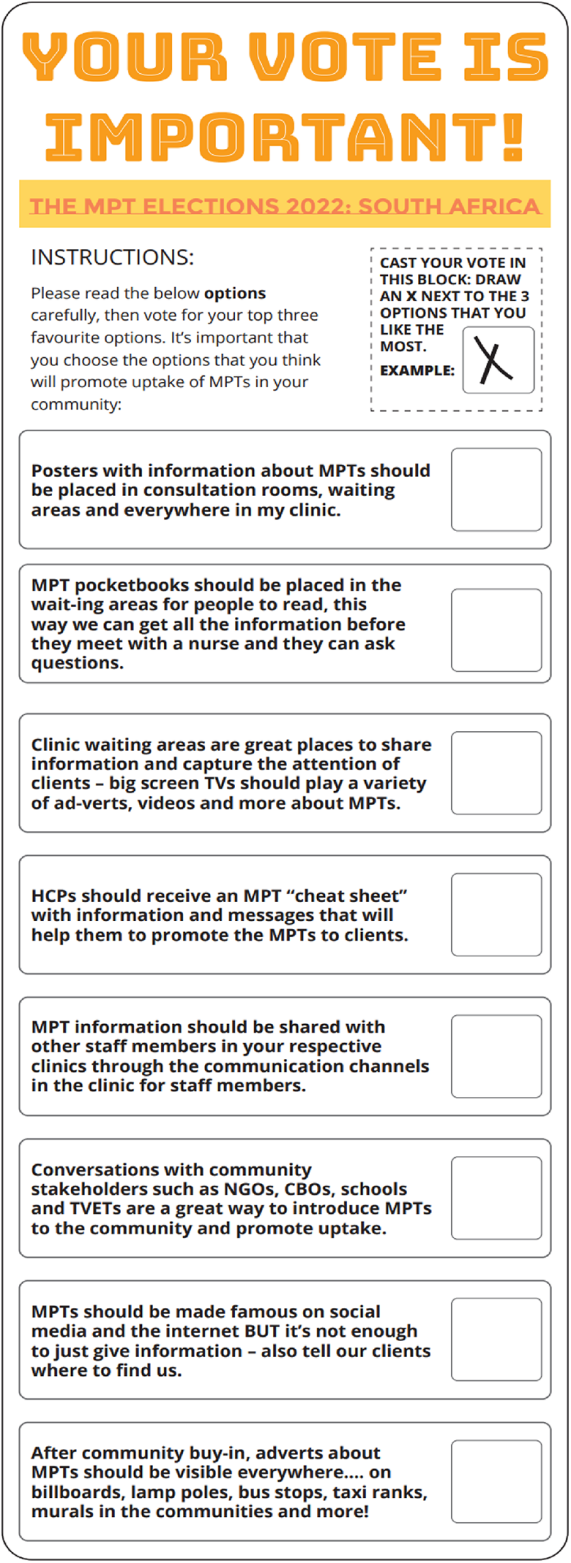
Healthcare provider voting ballot.

The demand creation elections activity aimed to explore which demand creation tactics would best support end-user uptake of HIV prevention methods (including new MPTs), and to gather insights and build evidence for developing demand creation and social mobilization approaches for future MPTs. The room was set up to simulate a real voting experience, with a voting booth, a voting box and posters. Participants were given a voting ballot paper containing eight potential demand creation tactics for raising MPT awareness in their community (Figure 1) and invited to vote for their top three tactics on the ballot. Following the voting, the results were tallied, and each tactic was discussed, allowing participants to share what they liked about the tactic and what they would change.

##### 2.3.1.2 Izikhokho zegame (skills & knowledge)

This activity aimed to establish the training needs of HCPs in relation to a potential MPT, including the resources needed and their preference for receiving training, skills building, and ongoing mentoring. Each HCP was given the opportunity to develop their ideal training package made up of five broad thematic areas (theory, assessment, practical, training others, and mentoring) using a card-sort activity. The training themes were divided into specific elements to illustrate self-learning, in-person learning, different assessment formats, different approaches to skills building and knowledge acquisition, and mentoring approaches.

**Figure 2:**
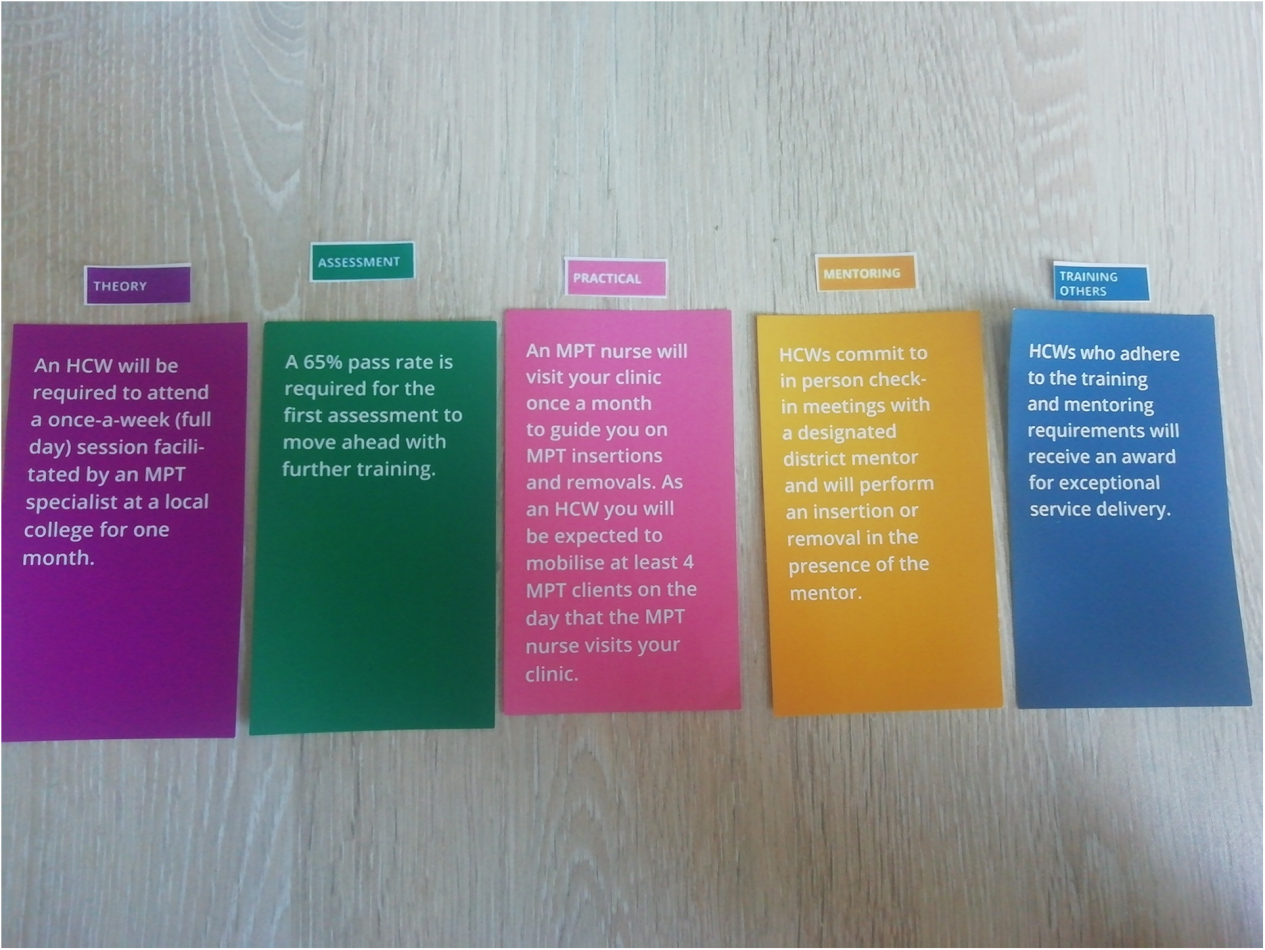
An example of *izikhokho zegame* (skills & knowledge) activity in action, a participants ideal training package.

Each participant was given five colour block cards with the five thematic areas written on them and four sub-element cards for each theme. On their own, participants read through each card and could choose one sub-element for each thematic area. At the end of the activity, each participant was left with a potential training package for MPT introduction that reflected their own needs. The facilitator then led a discussion based on the packages that they built. Figure 2 outlines an example of an ideal training package.

#### 2.3.2 Key informant IDIs

IDIs were conducted and audio recorded via MS Teams by one of the study team members (AK, PM, NM or JM) with master's degrees and trained in qualitative data collection methodologies, the study protocol and research ethics. A structured interview guide was used to guide the discussion, exploring key considerations in adopting new MPT methods, and reflecting on the 2014 contraceptive implant implementation process (14). Each IDI was up to 40 min in duration.

### 2.4 Data management and analysis

To ensure anonymity of the participants, each participant was allocated a unique identification number. All hard copies of completed data collection instruments were kept in locked storage cabinets, only accessible to the study team. Electronic data were stored on password-protected computers of the study team. Research activities were conducted predominantly in English however, sometimes, local languages were used by the participants.

#### 2.4.1 Healthcare provider workshops

The two or three observation guides from each workshop were transcribed by each observer and consolidated by either the Researcher (PM) or Associate Researcher (NM), to produce one consolidated observation guide per workshop for analysis. Data were double coded to ensure reliability of coding. Facilitated using NVivo software (33), data from the observation guides were coded deductively to describe and explore preferences for the different demand creation tactics and the training package sub-elements. The different demand creation tactics guided the coding for the demand creation elections. Content analysis (34) was used to analyse the data.

#### 2.4.2 Key informant IDIs

IDIs were transcribed by a fieldworker and then cross-checked for accuracy by the Associate Researcher (NM) and Research Intern (JM). One analysis workshop was held where four study team members (PM, NM, JM, and AK) reviewed the transcripts thematically, identified emerging themes and coded facilitated by the NVivo (33) software. The first round of codes was created through the open coding of one transcript during a workshop, to ensure reliability of coding. Then, using axial coding, these codes were organized into 13 codes. The remaining five transcripts were coded by two study team members (AK and NM). Once all the transcripts were coded, the 13 codes were further grouped into five major themes, namely: “*HCP training*”, “*messaging and demand creation*”, “*policy considerations*”, “*preparing for choice*”, and *“sustainability of MPT provision*”.

### 2.5 Ethical approvals and considerations

Ethical approval for the study was granted by the Human Research Ethics Committee (HREC) of the University of the Witwatersrand (M220305). Relevant provincial research approval was also granted. Written informed consent was obtained from participants before the workshop began and prior to the IDI. Participants received a signed copy of the informed consent form. HCPs were reimbursed ZAR50 ($2.75) for transport and ZAR200 ($10.95) for participating in the half-day workshop.

## 3 Results

### 3.1 Study sample

Twenty-one HCPs from across three provinces participated in PAR workshops (9 in OR Tambo, 8 in King Cetshwayo and 4 in Tshwane). All participants were Professional Nurses working at primary healthcare facilities and 90% (*n* = 19) were female.

Six IDIs were conducted with KIs: 4 Facility Managers, 1 NDoH representative, and 1 DoH Provincial Deputy Director. Participants were all females, ranging from 30 to 60+ years of age. All KIs had more than 10 years of experience in the field of SRH/HIV policy/advocacy/research.

### 3.2 HCP demand creation elections

The most voted for demand creation tactic for the MPT introduction was to have conversations with community stakeholders (including non-governmental organisations (NGOs), community-based organisations (CBOs), schools, and Technical and Vocational Education and Training (TVET colleges)) (22%, *n* = 14), followed by having MPT information in the clinic waiting areas (19%, *n* = 12) (Table 1). sixteen percent (*n* = 10) of HCPs thought that posters in the consultation rooms, waiting areas and walls of the clinic would be a good MPT demand creation tactic. Only 4 HCP participants (6%) liked the idea of MPT adverts visible everywhere, on billboards, lamp posts, taxi ranks or on murals in the community. None of the HCPs liked the idea of HCPs having a cheat sheet^1^ with MPT information and messaging on.

**Table 1:** Demand creation election results.

Demand generation tactic	Total	
	*N*	%
Conversations with community stakeholders (NGOs, CBOs, schools and TVETs) are a great way to introduce MPTs to the community and promote uptake.	14	22%
Clinic waiting areas are great places to share information and capture client attention—big screen TVs should play a variety of adverts, videos and more about MPTs.	12	19%
MPT posters should be placed in consultation rooms, waiting areas and everywhere in my clinic.	10	16%
MPTs should be made famous on social media and the internet but also tell our clients where to find us.	8	13%
MPT pocketbooks should be placed in the waiting areas, so patients can get all the information before they meet with a nurse and ask questions.	8	13%
MPT information should be shared with other staff members in the clinics through the communication channels in the clinic for staff members.	7	11%
After community buy-in, MPT adverts should be visible everywhere…. on billboards, lamp poles, bus stops, taxi ranks, murals in the communities and more!	4	6%
HCPs should receive an MPT “cheat sheet” with information and messages that will help them to promote the MPTs to clients.	0	0%
Total number of votes	63	100%

#### 3.2.1 HCP training requirements for the MPT introduction

Table 2 presents the results of the *Izikhokho zegame* activity, as voted by the 21 HCPs.

**Table 2:** The ideal MPT training package, as voted by HCPs.

Theme	Sub-element	Total votes	
		*N*	%
Theory	**HCWs will attend ONE in-person training in their district comprising of theoretical and practical sessions. A training manual with all MPT in-formation will be couriered to selected clinics.**	**11**	**52%**
	Complete an online theoretical course on the MPT. The course concludes with an assessment and if passed will secure your place for further training	6	29%
	An HCW will be required to attend a once-a-week (full day) session facilitated by an MPT specialist at a local college for one month.	2	10%
	HCWs are expected to work through this within a given time frame before completing an assessment.	2	10%
Assessment	**A 65% pass rate is required for the first assessment to move ahead with further training.**	**7**	**33%**
	**A 70% pass rate is required for the first assessment to move ahead with further training.**	**7**	**33%**
	A formal open book exam will be required as the first assessment with a time limit. The Clinic Manager will oversee the exam at the clinic and send the completed exam sheet to the organizers for formal marking.	4	19%
	An 85% pass rate is required for the first assessment to move ahead with further training.	3	14%
Practical	**HCWs that pass the speed test will receive a certificate of compliance. Thereafter invited to a week-long “on the job training’’ of how to insert and remove an implant**	**9**	**43%**
	An MPT nurse will visit your clinic once a month to guide you on MPT insertions and removals. As an HCW you will be expected to mobilize at least 4 MPT clients on the day that the MPT nurse visits your clinic.	5	24%
	Pass the minimum score and advance to attend an in-person workshop. The workshop will be a theoretical refresher of the training and focused on practical application. The training will be completed with an exam.	4	19%
	HCW will have to complete an assessment after completing the in-person training.	3	14%
Mentoring	**While studying and applying your knowledge at the clinic, you will have a dedicated mentor who will assist you with clinical enquiries. Submit daily reports to your mentor.**	**7**	**33%**
	The more you report your successes & challenges to your mentor, the better your chances of becoming a national trainer.	6	29%
	HCWs commit to in person check-in meetings with a designated district mentor and will perform an insertion or removal in the presence of the mentor.	6	29%
	HCWs commit to mentoring calls via WhatsApp at least once a month with a clinical mentor.	2	10%
Training others	**HCWs will be expected to use the training manual for training and provide mentorship to other HCWs on how to become fully fledged MPT nurses.**	**10**	**48%**
	HCWs who complete their training and meet their targets will have the honour of recruiting, training and mentoring 2 or more HCWs from their clinic on an ongoing basis.	5	24%
	HCWs who adhere to the training and mentoring requirements will receive an award for exceptional service delivery.	3	14%
	I really don't want to train others.	3	14%

##### 3.2.1.1 Theory

Over half preferred attending once-a-week in-person training (*n* = 11), with 29% (*n* = 6) indicating they preferred an online theoretical MPT course. The benefits of in-person training included the opportunity to engage with the course instructor and ask clarifying questions, which they felt would be harder online. Those that preferred completing a course online liked the convenience to complete the course in their own time. However, a few challenges were anticipated, these included access to internet data, internet connection challenges, and lack of time to do the training at home after hours. A one-day training was preferred over attending a session weekly. The HCPs' busy schedules meant working through the training alone would be hard.

##### 3.2.1.2 Assessment

Most workshop participants agreed (66%, *n* = 14) that an average of 65%–70% was an appropriate, and attainable, pass mark for the first assessment before doing further training, rather than a pass mark of 85% or writing an open-book exam. Participants indicated that an 85% pass mark was considered high for a new subject and felt that their workload would hamper them from achieving this mark. However, some mentioned that 85% was an appropriate score, using it as a good measure of HCPs understanding of the training content and capability to insert and remove the MPT implants. The open-book exam was viewed as time-consuming, as HCPs were preoccupied with work. Practically, they also mentioned that Clinic Managers would not be able to oversee an exam because they are busy.

##### 3.2.1.3 Practical

After passing the first assessment, most preferred a one-week, on-the-job training on insertion and removal of an MPT (43%, *n* = 9), compared to an exam, an additional assessment, or a monthly visit from an MPT nurse. Participants said that receiving certificates is personally important, providing proof that they are trained on MPT's, allowing them to advance in their careers. Generally, HCPs felt that attending practical training would help them become proficient at MPT implant insertion and removal, however, some HCPs were not in support of a week-long training.

##### 3.2.1.4 Mentoring

A third of participants (33%, *n* = 7) preferred having a dedicated mentor to assist with clinical enquiries, with daily reports to their mentor compared to monthly mentoring calls via WhatsApp with a clinical mentor, a designated district MPT mentor or frequent reporting to a mentor about the successes and challenges of MPT insertion. HCPs said that reporting daily to mentors would allow them to report strengths and weaknesses, helping them stay accountable and provide better MPT services. Where they struggled, the mentor could provide guidance and strategies for improvement. Other HCPs thought that daily reporting to a mentor would be demanding; they suggested reporting weekly, bi-weekly, or once a month. Some HCPs liked the idea of performing insertions and removals in the presence of their designated district MPT mentor, with the mentor correcting any mistakes, thereby improving their skills and confidence. Almost a third of HCPs (*n* = 6), liked the idea of becoming a national MPT trainer, as it allowed for career growth and recognition.

##### 3.2.1.5 Training others

Almost half of the HCPs (48%, *n* = 10) chose the option that required them to provide training with a training manual so others could also be capacitated to provide MPTs, once the products become available. Task sharing, by training and mentoring other nurses, was seen as important, avoiding tasks becoming one person's responsibility, providing cover when someone is on leave, and providing opportunity for training of new nurses. This could be done by an MPT champion in each clinic. A few HCPs mentioned that they would want recognition for adhering to training, mentoring, and providing exceptional service delivery. Only 3 HCPs did not want to train others, noting they didn't like training, and that it would be a huge responsibility.

### 3.3 Key considerations for future MPT implementation

The following five themes emerged during the KI IDIs as considerations for future MPT implementation: healthcare provider training, messaging, and demand creation, policy considerations, preparing for choice, and sustainability of MPT provision. Table 3 outlines the themes and sub-themes that were identified: the related quotes are embedded in the text.

**Table 3:** Key informant interview themes and sub-themes.

Theme	Sub-themes
Provider training	Provider training
Messaging & demand creation	Correct understanding
	Myths
	Peer educators
Policy considerations	Age of consent
	Distribution of condoms
	Priority populations & areas
	Safety and efficacy across a wide group of populations
Preparing for choice	Acceptability
	Choice
	Combination prevention
Sustainability	Few healthcare visits

#### 3.3.1 Healthcare provider training

All KIs indicated that training for HCPs is crucial because MPTs are new products and there is noted anxiety around new interventions. While there are many similarities between the MPT and contraceptive implant and some basic implant knowledge base, one KI noted that provider training should occur well ahead of MPT implementation, ensuring that providers feel well prepared for MPT insertion and removal, especially due to the novel aspects of the biodegradable and refillable MPT implants:

“*we’ll have to provide sort of training because uhh it will be a new uh thing for the providers … something that has never been done before. Obviously, … they do perform similar processes like you’ve just mentioned that the Implanon. So, … it will be easier for them to sort of uh shift, their knowledge of how to insert, insert the implant eh with the biodegradable and the non-bio because then even to remove the non-bio, they might just need to be orientated, …we know health care providers they get very anxious when new things come and they start having attitude it's because we don't prepare them enough. We need to make enough sessions for them to be prepared.*” (Female, 42)

“*Some of the professional nurses …were trained on inserting [Implanon], but the removal were not trained and they’ve got to ask, ask for the doctor or [tell] the client to go to other facilities for the removal… If you train the professional nurses on insertion [of the MPT implant], also train them removal*” (Female, 55)

Some noted that training manuals and the new products should be available at the training and that time should be allocated for practical demonstrations.

#### 3.3.2 Messaging and demand creation

Under this theme, participants discussed ensuring accurate knowledge and understanding about PrEP and the new MPT methods, including dispelling myths and preferred dissemination platforms.

##### 3.3.2.1 Accurate knowledge and understanding

KIs highlighted that accurate knowledge and understanding of any new method is crucial to ensure that potential end users can make an informed choice and use the method effectively.

“*enhance the capability of young people to broaden…their understanding of different methods so that they can choose based on their own … preferences and …what's convenient for them and what would work best for them as opposed to what my grandmother or my mother took.*” (Female, 60+)

Reflecting on the implementation of the contraceptive implant, some KIs noted that addressing local myths, especially those around side effects, with end users, families, and the local communities is a crucial step to ensure accurate product understanding. Furthermore, there must be timeous messaging to enable understanding and prevent myths.

“*If you don't reassure people around … the side effects and … what to expect, you know if it does come it's when you speaking to your neighbour speaking, to your mother is speaking to your colleagues or speaking to your friends and you getting all kinds of…information that may not be accurate.*” (Female, 60+)

“*the* [contraceptive] *implant is not likeable, people don't like it …if something new is coming you need to do a lot of grounds work of orientating people towards it because Implanon just came and people were inserted and then the myths started where they went home, and somebody told them, they're getting more fat.*” (Female, 42)

##### 3.3.2.2 Dissemination platforms

Information, education, and communication (IEC) materials need to be tailored for different age groups, ensuring that the community, as well as potential end users, are educated on these new products.

“*when you bring on new innovations…we don't take much time eh making them understand what is the benefits of…the [product] and how is it different from what has been there historically. So, we need to focus on that so that we can get the buy-in from both the providers and the beneficiaries*” (Female, 42)

Traditional IEC materials such as posters and pamphlets were mentioned as platforms for dissemination as well as video recordings and community radio. One KI noted that the National Department of Health should play a role in the dissemination of accurate information but noted that that online platforms like Tik Tok must be active and accurate. Another KI noted that the use of peer educators was a good strategy to reach young people.

“*I don't understand the language of the youth… we put the younger generation there so that when they talk, they talk one in the same language isn't it?*” (Female, 52)

#### 3.3.3 Policy considerations

When reflecting on the policy considerations of adding a new MPT method to the SRH prevention package, one KI (NDoH representative) outlined the following processes that would need to be followed: regulatory approval for the product, followed by a review from an expert committee in terms of cost-effectiveness. The South African Health Product Regulatory Authority (SAHPRA) reviews the safety profile, while National Essential Medicines List Committee conducts cost effectiveness, comparing what's available in the market. The guidelines then need to be amended or addenda added, including the specific initiation processes and standard operating procedures for that new product.

The age of consent and inclusion of specific population groups in the research were other policy considerations that were mentioned. This is to ensure that when the product is available, it is safe and effective for a wide range of population groups, specifically those who are under 18 years and pregnant women.

“*under 18s are excluded from the rings at the moment and …for CAB-LA* [long acting cabotegravir] *pregnant women are not included … and I think those … things became a barrier and …the product is already coming in with a shortcoming*.” (Female, 60+)

A few KIs noted that priority populations and geographic areas should be targeted when these products are provided. As with oral PrEP, the suggestion was to start in areas with the highest burden of HIV and unmet need for contraception and then expand to a wider areas/population.

“*we know kuthi [that] even the 11-year-old are sexually active. So, if you cannot give them PrEP, what [can you give*?].” (Female, 49)

“*we need to think through that carefully … using our population and incidence data. So, if we bringing in an MPT … that is … a combination of HIV prevention as well as contraceptives, we then need look at where is the greatest need.*” (Female, 60+)

#### 3.3.4 Preparing for choice

Among the KIs there was overall acceptability for the inclusion of MPT methods into the package of HIV and pregnancy prevention services, with an emphasis that new options provide greater choice:

“*We actually have multiple different options that people could choose uh as a contraceptive choice, uhm I think for … HIV prevention the more options we have the better and … the reason for that is that … we know that one size doesn't fit all. You know, we need to think about what the different preferences are… What people find convenient or what they find uh easy to use, or easy-to-access, will influence their choices.*” (Female, 60+)

“*There was a condom only for HIV prevention, but PrEP came then we were able to give people choice. So, it's always best if … people have a choice to different methods.*” (Female, 49)

Two KIs noted that these new products provide potential end users with additional choices for combination prevention, allowing us to “win” two battles, both unintended pregnancy and HIV.

“*Once we use … combination prevention methods and then eh we look into institutions of higher learning …and do activations in schools and…we get the injectables going we know that we have won, we might just go there … saying we are winning some kinds of battles*” (Female, 42)

However, one KI cautioned that the social context need to be considered when providing people with new contraception and HIV prevention methods, taking time to sensitize people on the new methods and how they differ from other methods, as historical choices impact future choices.

#### 3.3.5 Sustainability of MPT provision

Two KIs highlighted the need for proper planning, budgeting, monitoring and evaluation by the Government and partners to ensure sustained access to such methods. Product affordability, availability, and access were also noted as key components of sustainability, both linked to continued use.

“*We need to think of ways to sustain because what I often find is that a new project started and then falls off because we haven't explored to say what we'll what could cause uh uh the government not to afford, maybe affordability or maybe uhm what could but what would cause the program to fall….Systems must be in place, proper budget, proper planning, monitoring and evaluation.*” (Female, 42)

“*The availability of the stock will also increase sustainability.*” (Female, 55)

Furthermore, four KIs noted the long-acting benefit of the MPT resulting in fewer healthcare visits, would be an enabler to the continued use and reduced patient load in clinics, thereby increasing acceptability of the method among clients and providers, and improving its ongoing availability, uptake, and use.

“*people will always forget at some point … to take the treatment on a daily basis. So, … if there is a long-acting uhm like the CAB one and the other ones it is … like that might improve … uptake and also just improve their adherence because then the client doesn*’*t have to come to the facility like often to get the treatment*.” (Female, 42)

“ *Even if they move to another location at least they are moving with the Implanon. We are trying … to reduce the number of people coming* [to the facility] *because …we’re having shortages of staff. So, if they just put it for a year and then come back …for another year, I think we'll be able to … reach them once, and also reduce the number of people coming to the facilities.*” (Female, 49)

## 4 Discussion

This study aimed to determine HCP needs and gather key policy and programmatic considerations for the introduction of new MPTs into the SRH package in South Africa. HCP training, messaging and demand creation, policy considerations, preparing for choice, and sustainability of MPT provision are the five key programmatic implications to consider. These can be grouped into policy level, facility level and individual level considerations.

At a policy level, policy amendments and sustainability of MPT provision need to be considered. As policy leaders, the NDoH will be responsible for amendments to the existing PrEP policy and guidelines (35). The priority populations, geographic target areas and inclusion/exclusion criteria will be important considerations for the introduction of new MPT methods.

KIs highlighted the need for proper planning, budgeting, monitoring and evaluation, prior to the introduction of new products to improve the sustainability of these interventions and the continued provision of and access to MPTs. Creating and maintaining a sustainable intervention is a challenge faced by all health systems stakeholders, including politicians, funders, providers, insurers, policymakers, taxpayers and patients (36). Product affordability, availability and accessibility are key elements of sustainability, both linked to continued and effective use. A meeting with key policy makers could be set up, to share these research findings.

At a facility level, HCP training need to be considered. Insights into HCP training needs, and preferred training methods were unpacked during HCP workshops. The ideal training package provides theoretical content through a face-to-face district level training, followed by a short assessment with a pass rate between 65%–70%. Those that pass, will then participate in a week of on-the-job training on how to insert and remove an MPT implant. A dedicated mentor would support clinical enquiries, while the HCP applies their knowledge in the clinic. Training and capacitating other HCPs would be a part of their role. KIs reinforced that HCP training needs to be ongoing and must include training on the removal of the implant, a finding supported by Adeagbo et al. (26). When reflecting on the lessons learned from the contraceptive implant implementation, Pleaner et al. (14) and Humphries et al. (37) both recommended capacity building for HCPs, including training on insertion and removal of implants: our research supports this finding. Due to the similarities between the contraceptive implant and the MPT implant, many HCPs will have existing knowledge as a basis for the MPT introduction. Reflections from oral PrEP implementation, suggest the training of various levels of HCP, not only those who are trained to provide anti-retroviral therapy (ART), to broaden access and relieve the existing burden on HCPs in the delivery of PrEP (38). Our research suggests alternative training strategies which were appealing to HCPs (certificate of compliance, dedicated mentor and fully fledged MPT nurse status). These research findings provide considerations for innovative approaches to HCP training, ensuring that we learn from previous implant introductions, to proactively address any pitfalls. Closer to the new MPT introduction, careful consideration is required by the NDoH, to ensure that there is adequate HCP training, planning, and preparation. Particular attention should be paid to HCP attitudes towards the new methods, given the bias of HCPs for specific methods (39). At the right time, engagements with HCPs and their representatives are key to building on our research findings.

At an individual level, messaging, demand creation and preparing for product choice need to be considered. The accurate knowledge and understanding of new MPT methods, through messaging and demand creation is crucial, particularly with stakeholders at a community level. A variety of demand creation methods (in-person, IEC materials, billboards and radio as well as online platforms) should be employed, seeking to reach as many people as possible. This is supported by Mataboge et al. (23). HCPs recognise that young people would like information in a language appealing to them, from someone who understands their needs, like a peer educator (40, 41). Myths and misconceptions, especially those that exist from the contraceptive implant implementation process (42–44) need to be addressed, as they may be a barrier to MPT implant introduction. In their research in Cape Town, Krogstad et al. (45) found that the uptake of contraceptive implants was declining, due in part to real experiences or myths about women being assaulted by robbers who physically remove the implants to smoke as drugs. In another study in the Western Cape and Gauteng, adolescent girls also mentioned this myth during focus group discussions (46). This reinforces the need for accurate information sharing and demand creation, especially about the composition of the implant. Mataboge et al. (23), presenting findings from another objective of this research study, highlights the various demand creation tactics and messages that were appealing to potential end users when MPT methods are closer to implementation. Further engagements with potential end users, local community groups and key stakeholders should be held to build on our research findings.

In 2023, South Africa welcomed the Dapivirine Vaginal Ring (47) into the package of PrEP options at specific demonstration sites (48) and anticipates the arrival of long acting cabotegravir (49). As part of this, work is underway, preparing people for a choice of methods. Counselling for choice, ensuring that people use their voice and agency to make the choices that meet their contraceptive needs (50), becomes increasingly important as the choices for contraception and HIV prevention expand.

### 4.1 Strengths and limitations

This study included participants from three South African provinces, and different cadres within the DoH, enabling a variety of perspectives to be represented. However, the KI response was low, resulting in a smaller IDI sample size is smaller than originally planned. Although the majority of participants were females, this sex-skew is representative of the country's HCP population, who are mostly female. We adopted novel methodologies in this study, allowing for triangulation of data.

Observations conducted during the workshops rely on the purposive selection of what information is important to note down. Each observation already contains an element of interpretation of what is important, therefore introducing bias (33). While valuable insights were provided, the HCP workshops relied on a small sample size and therefore the results may not be nationally representative but provide insight to HCPs perceptions across different areas. Also, our research only included HCPs who were professional nurses, however many other cadres are involved in provision of SRH services, and they may hold different opinions on appropriate training methods and their perceptions on demand creation and messaging.

## 5 Conclusion

There are many lessons to be learned from the contraception implant and oral PrEP implementation that can be proactively considered when preparing for MPT introduction. HCP training and messaging and demand creation are of particular importance, ahead of MPT introduction. Providing this feedback to policy makers is key to ensure these lessons are learned and proactively addressing them will improve the success of the new MPT introductions.

## Data Availability

The raw data supporting the conclusions of this article will be made available by the authors, upon reasonable request.

## Footnotes

^1^Also known as a crib sheet, with a concise set of notes, used for quick reference
